# Supplementary material for: Sorting at embryonic boundaries requires high heterotypic interfacial tension
Source: Nat Commun. 2017 Jul 31;8:157. doi: 10.1038/s41467-017-00146-x (PMC5537356; doi:10.1038/s41467-017-00146-x)
Supplement: Supplementary file 2 — Supplementary Software 1 [file 41467_2017_146_MOESM2_ESM.zip › PottsModel/SrcPottsModel/doc/model/class-use/Lattice.html]

Uses of Class model.Lattice


JavaScript is disabled on your browser.


Skip navigation links


- Overview
- Package
- Class
- Use
- Tree
- Deprecated
- Index
- Help

- Prev
- Next

- Frames
- No Frames

- All Classes

## Uses of Class model.Lattice

- Packages that use Lattice

  | Package | Description |
  | --- | --- |
  |  |  |
  | --- | --- |
  | engine |  |
  | gui |  |
  | model |  |
- - ### Uses of Lattice in engine

    Fields in engine declared as Lattice

    | Modifier and Type | Field and Description |
    | --- | --- |
    |  |  |
    | --- | --- |
    | `Lattice` | PottsEngine.`aLattice` |

    Constructors in engine with parameters of type Lattice

    Constructor and Description

    `PottsEngine(Constants pConstants,
    Lattice pLattice)`
  - ### Uses of Lattice in gui

    Methods in gui with parameters of type Lattice

    | Modifier and Type | Method and Description |
    | --- | --- |
    |  |  |
    | --- | --- |
    | `void` | SnapshotManager.`initializeMatrix(Lattice pLattice)` |
    | `void` | PottsCanvas.`initializeMatrix(Lattice pLattice)` |
    | `void` | SnapshotManager.`repaint(Lattice pLattice)` |
    | `void` | SnapshotManager.`saveSnapshot(Lattice pLattice, java.lang.String filename)` |
  - ### Uses of Lattice in model

    Methods in model that return Lattice

    | Modifier and Type | Method and Description |
    | --- | --- |
    |  |  |
    | --- | --- |
    | `static Lattice` | Lattice.`inverse(Constants pConstants)` |

    Methods in model with parameters of type Lattice

    | Modifier and Type | Method and Description |
    | --- | --- |
    |  |  |
    | --- | --- |
    | `double` | IEnergyFunction.`computeEnergyLocally(Lattice aLattice, Pixel pSource, Pixel pTarget)` Computes the energy of the two pixels and their neighbours. |
    | `double` | IEnergyFunction.`computeEnergyLocallyIfSwapped(Lattice aLattice, Pixel pSource, Pixel pTarget)` |
    | `static void` | LatticeUtilities.`writeLattice(Lattice l, java.lang.String path)` |

    Constructors in model with parameters of type Lattice

    Constructor and Description

    `Cell(Cell.CellType pType,
    int pID,
    Lattice pLattice)`

    `Cell(int pID,
    Lattice pLattice)`

    `HexagonPositionManager(Lattice pLattice)`

    `Pixel(int pX,
    int pY,
    Cell pC,
    Lattice pL)`

    `SquareCell(Cell.CellType pType,
    int pID,
    Lattice pLattice)`

    `SquareCell(int pID,
    Lattice pLattice)`

    `SquarePositionManager(Lattice pLattice)`

Skip navigation links


- Overview
- Package
- Class
- Use
- Tree
- Deprecated
- Index
- Help

- Prev
- Next

- Frames
- No Frames

- All Classes
